# Exploring the link between tooth loss, cognitive function, and brain wellness in the context of healthy aging

**DOI:** 10.1111/jre.13280

**Published:** 2024-05-06

**Authors:** Roger D. Newman‐Norlund, Santosh Kudaravalli, Anwar T. Merchant, Julius Fridriksson, Chris Rorden

**Affiliations:** ^1^ Department of Psychology, College of Arts and Sciences University of South Carolina Columbia South Carolina USA; ^2^ Department of Epidemiology and Biostatistics, Arnold School of Public Health University of South Carolina Columbia South Carolina USA; ^3^ Department of Communication Sciences and Disorders, College of Arts and Sciences University of South Carolina Columbia South Carolina USA

**Keywords:** brainage, cognition, MCI, Montreal cognitive assessment, MRI, oral health

## Abstract

**Aims:**

The aim of this study was to evaluate the utility of using MRI‐derived tooth count, an indirect and nonspecific indicator of oral/periodontal health, and brain age gap (BAG), an MRI‐based measure of premature brain aging, in predicting cognition in a population of otherwise healthy adults.

**Methods:**

This retrospective study utilized data from 329 participants from the University of South Carolina's Aging Brain Cohort Repository. Participants underwent neuropsychological testing including the Montreal Cognitive Assessment (MoCA), completed an oral/periodontal health questionnaire, and submitted to high‐resolution structural MRI imaging. The study compared variability on cognitive scores (MoCA) accounted for by MRI‐derived BAG, MRI‐derived total tooth count, and self‐reported oral/periodontal health.

**Results:**

We report a significant positive correlation between the total number of teeth and MoCA total scores after controlling for age, sex, and race, indicating a robust relationship between tooth count and cognition, *r*(208) = .233, *p* < .001. In a subsample of participants identified as being at risk for MCI (MoCA <= 25, *N* = 36) inclusion of MRI‐based tooth count resulted in an *R* ^2^ change of .192 (*H*_0_ = 0.138 → *H*_1_ = 0.330), *F*(1,31) = 8.86, *p* = .006. Notably, inclusion of BAG, a valid and reliable measure of overall brain health, did not significantly improve prediction of MoCA scores in similar linear regression models.

**Conclusions:**

Our data support the idea that inclusion of MRI‐based total tooth count may enhance the ability to predict clinically meaningful differences in cognitive abilities in healthy adults. This study contributes to the growing body of evidence linking oral/periodontal health with cognitive function.

## 1 INTRODUCTION

Alzheimer's disease (AD) and related dementias (ADRD) represent a massive societal challenge as lifespan continues to increase worldwide.^1^ The prevalence of ADRD is expected to reach 139 million by 2050,^2^ with an estimated societal burden of over 2.2 trillion dollars per year by 2060.^3^ Besides immense societal costs, the cognitive impairments associated with ADRDs can lead to difficulties in daily living activities,^4^, ^5^ communication and language,^6^, ^7^, ^8^ and decision‐making.^9^, ^10^ In advanced stages, dementia often results in complete loss of independence^11^ and significant decline in quality of life for both the individuals affected and their caregivers,^11^, ^12^ rippling through families and communities, posing significant emotional, financial, and social challenges.^13^

Given the enormous economic, societal, and individual costs of ADRD, early identification of ADRDs, particularly at the prodromal stage of mild cognitive impairment (MCI), is crucial.^14^ In its early stages MCI is characterized by a noticeable decline in cognitive abilities, including memory and thinking skills, but these deficits have not yet progressed to the point at which they interfere with activities of daily living (ADLs).^15^ Research indicates that a substantial proportion of MCI cases progress to AD or dementia, in some cases as many as 18.4%,^16^ making early detection vital for timely intervention and management.^17^ Currently, predictive models of MCI and MCI progression rely on a range of variables, including health information derived from standard cognitive tests like the Montreal Cognitive Assessment (MoCA)^18^ and Mini‐Mental State Examination (MMSE),^19^ digital wearable technologies such as monitoring of bodily movement,^20^ fluid biomarkers,^21^ and brain imaging markers.^22^, ^23^ Recently brain age, an MRI‐derived metric of overall brain health which uses machine learning to estimate an individual's age based on a single T1‐weighted MR image, has emerged as a potential predictor of health and cognition in both healthy and clinical populations.^19^, ^24^ Based on structural integrity, the publicly available brainageR pipeline estimates a person's age, and the difference between this estimated age and a person's chronological age is termed the brain age gap (BAG).^25^ BAG has been associated with various clinical conditions and aging processes and studies have shown that a larger brain age gap can indicate accelerated aging or the presence of neurodegenerative conditions.^24^, ^26^

In addition to medical imaging‐based metrics such as BAG, recent studies report robust relationships between various measures of oral/periodontal health and cognitive impairments like MCI and dementia.^27^, ^28^, ^29^ These findings suggest that variables related to oral/periodontal health, such as engagement with dentists, gum disease, tooth loss, and oral hygiene, have the potential to offer valuable insights into cognitive decline.^30^ Considering the relatively clear data supporting the relationship between oral health and cognition, it is likely that models predicting early MCI can be improved by including these data. However, current predictive models for MCI seldom incorporate such information. This omission represents a significant gap in research, as given the relative ease and cost‐effectiveness of extracting basic metrics of oral health via inspection of dental records, oral examinations, and patient‐reported outcomes.

Inclusion of data related to oral/periodontal health data in predictive models could be used to refine and enhance the predictive accuracy of MCI models and there is a clear need to evaluate the relative utility of various types of data in such models. The current study examined the relative value of MRI‐derived markers of brain health (BAG) and an MRI‐derived indirect proxy of oral/periodontal health (total tooth count) as predictors of cognition. Using data from a large, multimodal repository on healthy aging (University of South Carolina's Aging Brain Cohort Repository), we systematically evaluated the relationship between MoCA scores, BAG, and total tooth count. Based on prior research highlighting the significance of both brain and oral/periodontal health in cognitive functioning, we predicted that the incorporation of both MRI‐based metrics would significantly enhance the accuracy of regression models predicting cognition.

## 2 METHODS

### 2.1 Design

A cross‐sectional quantitative research design using self‐reported sociodemographic and periodontal/oral health surveys, MRI‐derived tooth count, and brainage and MoCA scores obtained by an NIH‐toolbox trained staff member was employed. This study adhered to STROBE (Strengthening the Reporting of Observational Studies in Epidemiology) guidelines of reporting.

### 2.2 Participants

Data was drawn from the Aging Brain Cohort (ABC) Repository study, a University of South Carolina‐funded cross‐sectional study investigating the interaction between health, lifestyle, cognition, and the brain in healthy aging, between its inception in September 2019 and December 2023. In this study, the sample size was determined by the availability of data from the repository associated with this study. All available data was used. As part of their participation in this study, participants underwent neuropsychological testing, filled out health‐related surveys, and submitted to an MRI examination in which high‐resolution T1‐weighted structural MRI images were acquired. This study was approved by the local University of South Carolina IRB. In order to enroll in this study, participants were required to meet the following criteria: Age between 20 and 80 years old at the time of testing, the ability to provide written informed consent, MRI compatibility (i.e., no metal implants, not claustrophobic, etc., and able to lie in a supine position for 1 hour, maximum girth <60in and maximum weight < 400 lbs), no acute or chronic conditions that would limit the ability to participate, no current severe illnesses (cancer), no unmanaged psychological diagnosis (i.e., schizophrenia or severe mood disorder), no current or past fatiguing illness or other major morbidities. Sociodemographic descriptives for this cohort can be found in Table 1.

**TABLE 1:** Descriptive data for demographic and health variables of interest.

Category	Measure	Valid	Miss/Unus	M ± (SD)	Min	Max	Range
Demographics	Race	329	0	W (269), AA (40), AS (10), NAPI (6)			
	Sex	326	3	*N* (M) = 83, *N* (F) = 244			
	Age	326	0	46.15 ± 18.31	20	80	60
	SES	311	18	1.16 ± 0.70	0	2	2
Cognition	MoCA Total Score	311	18	27.23 ± 2.58	14	30	16
	Memory Index Score	328	1	13.53 ± 2.40	1	15	14
	Executive Index	328	1	11.86 ± 1.25	6	13	7
	Attention/Concentration Index	328	0	16.79 ± 1.70	0	18	18
	Language Index	329	0	5.40 ± 0.88	0	6	6
	Visuospatial Index	329	2	6.44 ± 0.81	3	7	4
	Orientation Index	327	1	5.94 ± 0.36	1	6	5
Oral health	Upper tooth count	196	133	13.25 ± 2.27	0	16	16
	Lower tooth count	189	140	13.68 ± 2.32	0	16	16
	Total tooth estimate	208	101	26.64 ± 3.83	0	16	16
Brain health	Brain age gap (BAG)	300	29	−3.01 ± 6.74	44.34	−23.91	20.44

Abbreviations: AA, African American; AS, Asian; Miss/Unus, missing data or distorted data rendering tooth enumeration impossible; MoCA, Montreal Cognitive Assessment (MCI, *N* = 36, non‐MCI, *N* = 160); NAPI, Native American/Pacific Islander; SES, socioeconomic status; W, White.

### 2.3 Cognitive Health Assessments (MoCA)

The Montreal Cognitive Assessment (MoCA) is a widely used cognitive screening tool designed to assess various cognitive domains, including memory, attention, language, visuospatial abilities, and executive function.^31^ It is often used by healthcare professionals to detect cognitive impairment or changes in cognitive function in individuals and the MoCA is particularly useful in the evaluation of mild cognitive impairment (MCI) and early stages of dementia, such as Alzheimer's disease.^31^, ^32^, ^33^ The MoCA results in scores for Orientation (6 points), Visuospatial Abilities (5 points), Naming (3 points), Attention and Concentration (6 points), Language (6 points), Abstraction (2 points). Delayed Recall (5 points) for a total possible score of 30 points, with higher scores indicating better cognitive function. A score below a certain threshold (often around 25) may suggest the presence of cognitive impairment.^31^

### 2.4 Oral/periodontal health questionnaire

The oral/periodontal health questionnaire consisted of eight self‐reported questions related to oral/periodontal health (see Table S1). Six of the questions were identical to those proposed by the Centers for Disease Control and the American Academy of Periodontology (CDC‐AAP) and an additional two were previously used in the Australian National Study of Oral Health (NSAOH).^34^ Therefore, our statistical analysis considered correlations between key variables and responses to each question (Q1‐Q8) individually.

### 2.5 MRI‐based tooth count

**FIGURE 1:**
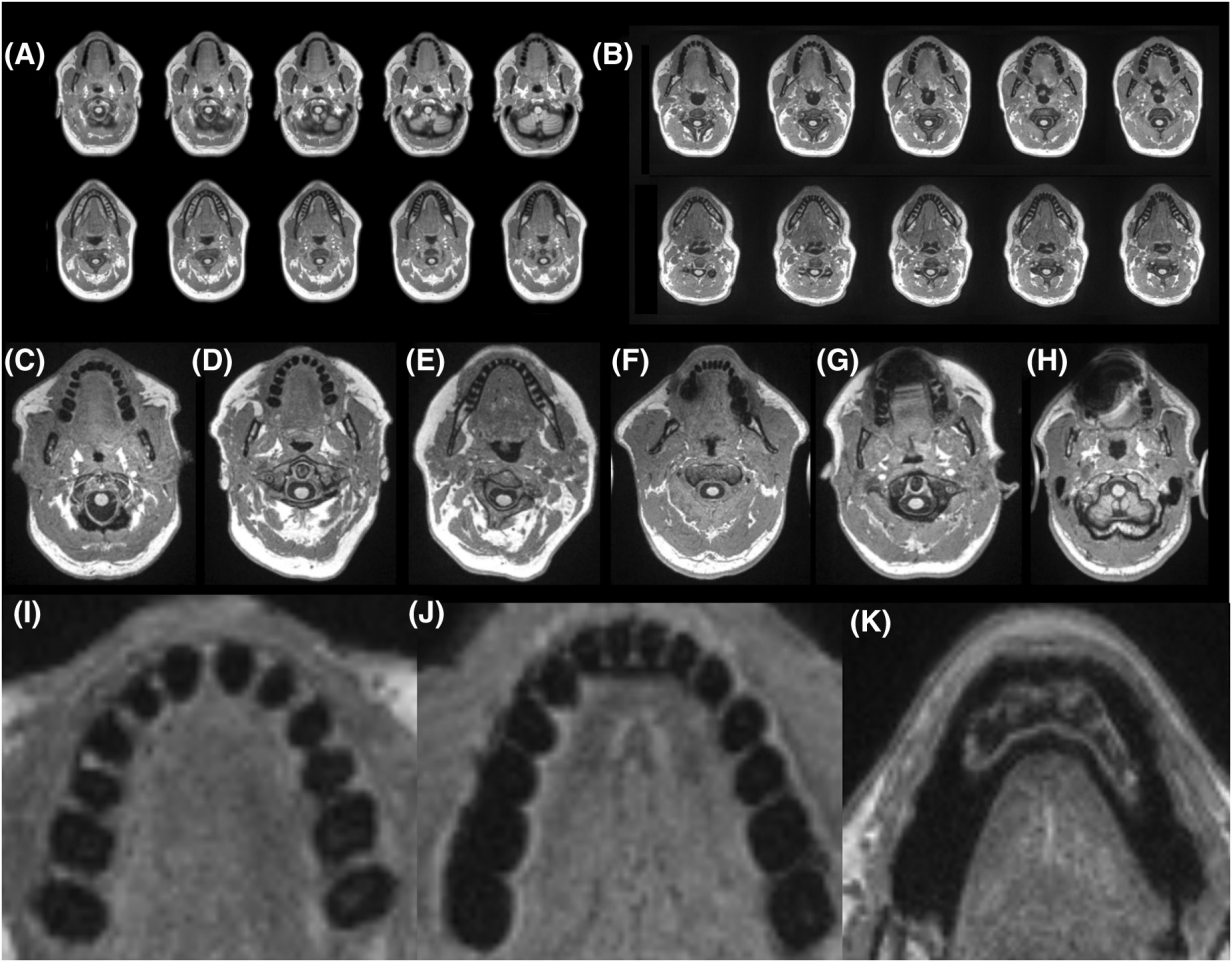
Teeth were manually counted based on visual inspection of axial images extracted from T1‐weighted MRI scans. (A, B) Sequential axial slices showing the upper (top row) and lower (bottom row) jaw for two individual participants without any MRI distortion and for whom calculation of upper and lower teeth was completed. (C) Example key axial image from a participant with 14 enumerable upper teeth (D) Example key axial image from a participant with 12 enumerable upper teeth. (E) Example key axial image from a participant with 16 enumerable lower teeth. (F) Minimal bilateral distortion caused by implants in the left and right lower teeth. (G) Significant midline distortion caused by a retainer affecting upper and lower jaw. (H) Significant distortion in the area of the upper teeth caused by dental bridge. (I–K) Visualization software (MRIcroGL12, https://github.com/rordenlab/MRIcroGL) allowed raters to zoom in on axial slices while scrolling in the superior/inferior direction, thus making tooth enumeration easier.

All participants underwent the same MRI scanning protocol on a 3 T Siemens PRISMA scanner with a 20‐channel head coil. T1‐weighted images were used for brain age estimation and were acquired using the following parameters: T1‐weighted imaging (MP‐RAGE) sequence with 1 mm isotropic voxels (e.g., the 3D image of the head was composed of 1 mm x 1 mm x 1 mm voxels), 256 × 256 resolution, 9° flip angle, and 192‐slice sequence with repetition time (TR) = 2250 ms, inversion time (TI) = 925 ms, and echo time (TE) = 4.11 ms. Manual teeth counts were derived from these T1‐weighted MRI images via visual inspection. Example T1‐weighted MRI images are shown in Figure 1. Specifically, two trained raters (RNN, SN) manually inspected these MRI images and counted the teeth in each quadrant of the mouth (upper left, upper right, lower left, and lower right). Proper recognition of teeth required more than just looking at individual images. Specifically, RNN and SN scrolled through MRI image sections containing teeth, in the superior and inferior directions, and it was helpful to follow the contours of suspected teeth through these levels to confirm that they were indeed teeth.

In cases of disagreement, raters discussed the images and agreed upon a final value. Tooth count was only discussed in cases where these MRI images were free of distortion due to MR artifacts caused by braces, dental implants, or other devices. In some cases, confounding signal distortion was limited to either the upper or lower jaw. In these cases, the unaffected level was judged (i.e., the upper teeth were scored in case of lower distortion). Dependent variables included *total upper teeth*, *total lower teeth*, and *total teeth*. Total teeth were calculated by adding the number of *upper teeth* and *lower teeth*. In cases where either the upper or lower teeth were obscured by MRI artifact we calculated total teeth as two times the number of teeth in the undistorted jaw level (i.e., in the case of upper distortion, *total teeth* was estimated as [*2 × total lower teeth]*). Because the direction and significance of model comparisons run with *total upper teeth*, *total lower teeth*, and *total teeth* were the same, we focus on *total teeth* as the primary indirect measure of oral/periodontal health.

Teeth were manually counted based on axial images extracted from T1‐weighted MRI scans. Sequential axial slices showing the upper (top row) and lower (bottom row) jaw for two individual participants without any MRI distortion and for whom calculation of upper and lower teeth was completed. (C) Example key axial image from a participant with 14 enumerable upper teeth (D) Example key axial image from a participant with 12 enumerable upper teeth. (E) Example key axial image from a participant with 16 enumerable lower teeth. (F) Minimal bilateral distortion caused by implants in the left and right lower teeth. (G) Significant midline distortion caused by a retainer affecting upper and lower jaw. (H) Significant distortion in the upper teeth caused by dental bridge (Figure 1).

### 2.6 Brain health estimate

Our primary measure of brain health was calculated using *brainageR*, a computer algorithm based on deep‐learning that has been shown to be both valid and reliable in past research relating brain health to a variety of clinical conditions including conversion from MCI to dementia.^35^, ^36^ Each participant's brainage value was calculated by entering their T1‐weighted image into the brainageR program which was a pre‐trained model created using >3000 healthy brains ranging from age 20 through age 80 (https://github.com/james‐cole/brainageR). This program relied on FSL Version 6.0.5,^37^ SPM12 version 7771 (https://www.fil.ion.ucl.ac.uk/spm/), and the Matlab 2023a toolbox. Brain health was operationalized as the brain age gap (BAG), that is, difference between an individual participant's chronological age and the estimated age of that participant based on their brain MRI data (i.e., BAG = *estimated brainage* – *chronological age*) such that higher numeric values reflected brains that looked “older than expected” and lower numbers reflected brains that looked “younger than expected.”

### 2.7 Statistical methods

Demographics and health were described by calculating means and standard deviations for continuous variables and percentages for categorical variables. An initial descriptive analysis assessed correlations between various demographic data and our primary dependent variables of interest, that is, total MoCA score, total number of upper teeth, total number of lower teeth, total teeth, oral/periodontal health questionnaire total scores, and brain age gap (BAG) (Table 1). We additionally quantified the frequency of MRI distortion that precluded enumeration of teeth in our sample.

Next, we examined the relationship between our primary measure of cognition, scores on the MoCA test, and both survey‐based and MRI‐based measures of total tooth count (Table 2). We additionally report results from a series of exploratory, uncorrected, post‐hoc Pearson's correlations between each of the MoCA component subscores and MRI‐based total tooth count metrics revealed robust correlations between total tooth count and scores on all individual subcomponents of the MoCA (Table 3).

**TABLE 2:** Pearson's correlations for demographic and variables of interest.

Variable	value	Age	Sex	Race	Econ
MoCA Total Score	*n*	328	328	307	282
	Pearson's *r*	−.367	.002	−.431	−.001
	*p*‐value	**<.001**	.97	**<.001**	.985
Upper Teeth	*n*	197	197	191	179
	Pearson's *r*	−.392	.032	−.283	.075
	*p*‐value	**<.001**	.66	**<.001**	.316
Lower Teeth	*n*	190	190	183	174
	Pearson's *r*	−.317	.094	−.368	.107
	*p*‐value	**<.001**	.198	**<.001**	.159
Total Teeth	*n*	209	209	202	191
	Pearson's *r*	−.387	.075	−.365	.097
	*p*‐value	**<.001**	.282	**<.001**	.184
Brain Age Gap (BAG)	*n*	301	301	283	263
	Pearson's *r*	−.316	.2	−.054	−.184
	*p*‐value	**<.001**	**<.001**	.363	.003

*Note*: Correlations between MoCA scores, Total Teeth, BAG, and responses to individual items on the oral/periodontal health questionnaire (Q1‐Q8) and MRI‐derived markers of oral and brain health. Correlations between basic demographic variables and the primary dependent variables of interest. Significant correlations in bold.

Abbreviations: MoCA, Montreal Cognitive Assessment, *n* = sample size.

**TABLE 3:** Correlations between MoCA subscores, tooth count, brainage gap, and Periodontal Health Questionnaire responses.

Variable		MoCA Total	Memory	Executive	Att/Conc	Language	Visuospatial	Orientation
Total Teeth	n	205	205	205	205	205	205	205
	Pearson's *r*	.248	.135	.099	.14	.147	.203	.258
	*p*‐value	**<.001**	.055	.16	**.048**	**.036**	**.004**	**<.001**
Brainage Gap (BAG)	*n*	297	297	297	297	297	296	297
	Pearson's *r*	−.011	−.014	−.007	−.014	.017	−.044	.077
	*p*‐value	.857	.807	.911	.807	.771	.455	.188
Q1: Gum disease	n	158	158	158	159	159	158	158
	Pearson's *r*	−.064	−.051	.004	−.044	−.042	−.06	−.011
	*p*‐value	.429	.527	.956	.589	.599	.457	.892
Q2: Lost bone	*n*	158	158	158	159	159	158	158
	Pearson's *r*	−.056	.081	−.123	−.073	−.115	−.021	.093
	*p*‐value	.492	.314	.128	.364	.153	.796	.249
Q3: Gum treatment	*n*	158	158	158	159	159	158	158
	Pearson's *r*	.103	.124	.043	−.025	.046	−.006	.159
	*p*‐value	.203	.123	.592	.758	.57	.941	.048
Q4: Lost teeth	*n*	158	158	158	159	159	158	158
	Pearson's r	.137	.101	.049	−.02	.1	.103	.057
	*p*‐value	.09	.21	.542	.801	.213	.203	.479
Q5: Mouthwash frequency	*n*	156	156	156	157	157	156	156
	Pearson's *r*	−.023	−.028	.007	−.031	.002	−.032	.04
	*p*‐value	.773	.729	.928	.705	.981	.693	.619
Q6: Flossing frequency	*n*	157	157	157	158	158	157	157
	Pearson's r	.098	−.067	.129	.177	.126	.151	−.047
	*p*‐value	.227	.408	.11	**.027**	.117	.062	.567
Q7: Gum health	*n*	158	158	158	159	159	158	158
	Pearson's *r*	−.143	.043	−.211	−.093	−.123	−.154	.068
	*p*‐value	.076	.595	**.008**	.248	.126	.055	.402
Q8: Bad tooth (3 mo.)	*n*	158	158	158	159	159	158	158
	Pearson's *r*	−.008	−.062	.021	.007	.055	.003	−.024
	*p*‐value	.924	.441	.793	.934	.496	.972	.764

*Note*: Conditioned on variables: sex, age, race. Correlation between MoCA sub‐scores, responses to individual items on the oral/periodontal health questionnaire (Q1‐Q8), brainage, and tooth count measures. Significant correlations in bold. Correlations between scores on MoCA Total and MoCA subtest scores and each of the following: (1) MRI‐derived of total tooth count, (2) MRI‐derived Brainage Gap (BAG), (3) Responses to individual items on the oral/periodontal health questionnaire (Q1‐Q8, see Tables S1–S5) after controlling for age, sex, race, and socioeconomic status.

A primary goal of this study was to evaluate the value of including MRI‐derived tooth count data in linear regression models predicting total MoCA scores. To test this, we conducted an ANOVA comparing two separate linear regression models. The null model (*H*_DEM_) used the total MoCA score as the dependent variable and demographic data (age, sex, race, and socioeconomic status) as predictor variables. Scores on the oral/periodontal health questionnaire were not included due to low correlations with the MoCA and reduction in sample size when including them. The second model (*H*_DEM+TEETH_) was identical to the first, except that it included the MRI‐based tooth metric total teeth as an additional predictor variable.

In order to evaluate improvements in model fit resulting from the addition of brain health related data to linear regression models predicting MoCA, we repeated the ANOVA described above, substituting BAG for tooth count (i.e., the null model (*H*_DEM_) contained demographic data and the second model (*H*_DEM+BAG_) additionally contained BAG).

Finally, in order to examine differential effects in low and high cognition groups, we divided our sample into two groups, MCI (*N* = 36) and non‐MCI (*N* = 160), based on established guidelines regarding the relationship between MoCA total scores and MCI risk established by Nasreddine and colleagues.^31^ Specifically, the MCI group consisted of participants with MoCA total scores <= 25 and the non‐MCI group included participants with MoCA total scores >25. Within each of these two groups (MCI, non‐MCI), we repeated ANOVAs comparing models based on demographic data (*H*_DEM_) to those including tooth count data (*H*_DEM+TEETH_).

## 3 RESULTS

### 3.1 Demographics and variables of interest

Basic demographic data were acquired from the entire population sample. This included age, race, sex, and socioeconomic status (low/med/high). Demographic and descriptive data regarding variables of interest for the entire sample are shown in Table 1.

### 3.2 Dental MRI data

Out of 329 total participants, T1‐weighted MRI data was present for a total of 308 participants (Figure 1). Out of these 308 scans, 36 T1‐weighted MRI scans did not cover the mouth area due to positioning in the scanner and another 64 T1‐weighted MRI scans (20.78%) exhibited distortion in both upper and lower jaws rendering them unusable for the purposes of enumerating the teeth. Twelve participants (3.90%) had MRI distortion limited to the upper jaw, and MRI distortion was only present in the lower jaw for another 19 (6.17%) participants, resulting in a total of 31 cases in which only the top teeth or only the bottom teeth were enumerable. In these cases, the number of total teeth was estimated by multiplying the number of countable teeth (either in the lower or upper jaw) by 2. Total teeth were estimated in this way for a total of 31 out of the total 208 usable cases.

A series of planned Pearson's correlations revealed that MoCA total scores, upper tooth count, lower tooth count, and total teeth were all negatively correlated with both age and race (lower in African Americans relative to Caucasian). Brain age gap (BAG) was significantly and positively associated with both age and sex (higher in Males than in Females). Total scores for oral/periodontal health were not significantly correlated with any demographic variables. Based on these results we controlled for age, sex, and race in subsequent reported correlation analyses (Table 1).

A second series of planned, one‐tailed Pearson's correlations, controlling for age, race, and sex, demonstrated a significant correlation between MoCA scores and number of upper teeth, *r*(197) = .212, *p* < .001, the number of lower teeth, *r*(190) = .263, *p* < .001, and estimated number of total teeth, *r*(208) = .233, *p* < .001 (Table 2).

A series of exploratory post‐hoc Pearson's correlations between various MoCA total and subscores and MRI‐based tooth count confirmed the presence of robust correlations between total teeth and scores on all the individual subcomponents of the MoCA (all *p*'s < .01) except Memory (*p* = .06) and Executive function (*p* = .16) (Table 3). Examination of the correlation between individual oral/periodontal health questionnaire items (Q1‐Q8) and MoCA scores revealed no relationships surviving after adjusting for the additional 56 comparisons (*p*_adjusted_ = .05/56 = .0008).

When predicting MoCA scores the demographic model which included age, sex, race, and socioeconomic status (*H*_DEM_) was significant, *F*(5,193) = 16.33, *p* = .001, with age and race having the most heavily weighted coefficients. The second model (*H*_DEM_ + TEETH), which included total teeth, was also significant, *F*(4,192) = 15.966, *p* < .001, with age, race, and total teeth emerging as the most significant individual predictors. Critically, the addition of MRI‐derived tooth count data as a predictor variable resulted in an *R* ^2^ change of 0.031 (*H*_DEM_ = 0.253 → *H*_DEM_ + TEETH = 0.294) which represented a statistically significant change in model fitness, FCHANGE (1192) = 11.070, *p* = .001 (Table S2).

In the model in which we substituted BAG for total teeth, the demographic model which included age, sex, race, and socioeconomic status (*H*_DEM_) was significant, *F*(4,282) = 22.02, *p* < .001, with age and race having the most heavily weighted coefficients. The model including BAG (*H*_DEM_ + BAG) was also significant, *F*(5,282) = 17.552, *p* < .001, with age and race emerging as the most significant individual predictors. The addition of BAG as a predictor variable did not change *R* ^2^ change of .00 (*H*_DEM_ = 0.241 → *H*_DEM_ + BAG = 0.241), thus there was no statistically significant change in model fitness, FCHANGE (1277) = 0.004, *p* = .94 (Table S3).

When constraining our analysis to the MCI group (MoCA scores <= 25, *N* = 36), the addition of MRI‐derived tooth count data to linear regression model containing age, sex, race, and socioeconomic status resulted in a significant model improvement and an associated *R* ^2^ change of .192 (*H*_DEM_ = 0.138 → *H*_1_ = 0.330), *F*(1,31) = 8.86, *p* = .006 (Table S4). Within the non‐MCI group (*N* = 160), the addition of MRI‐derived tooth count data as a predictor variable failed to improve model fit, with an *R* ^2^ change of .010 (*H*_DEM_ = 0.088 → *H*_DEM_ + TEETH = 0.98), *F*(1,155) = 1.62, *p* = .21 (Table S5). Evaluation of the effects of adding BAG to linear regressions models of MoCA scores in the high (>25) and low (<=25) MoCA groups revealed that model fit was not improved by inclusion of BAG gap scores, *F*(219,1) = .015, *p* = .903 and *F*(52,1) = 2.96, *p* = .091, respectively, all *R* ^2^ change <.015.

## 4 DISCUSSION

Our analyses suggest that MRI‐derived tooth count predicts cognition better than a currently available MRI‐based measure of overall brain health (i.e., BAG), especially in individuals at risk for converting to MCI or dementia (MoCA <= 25). MRI‐derived tooth count may be particularly useful to clinicians interested in characterizing cognition. The current study investigated the relationship between cognition, brain health, and total tooth count, and an MRI‐based indirect and nonspecific indicator of oral/periodontal health in a cohort of healthy adults ranging from age 20 to age 80. Based on prior empirical evidence we predicted that MRI‐based metrics of brain health (BAG) and total tooth count would both provide valuable information in models that predict cognitive status, even after controlling for demographic variability in age, sex, race, and socioeconomic status. Within our sample, total tooth count was found to be independently related to cognition. This was especially the case in individuals with MCI (MoCA <= 25, *N* = 36). Contrary to our expectations, overall brain health, as measured by BAG, was not independently associated with cognitive abilities as assessed with the MoCA.

These results are novel for a number of reasons. First, this study demonstrates the feasibility of extracting an oral/periodontal health proxy from research quality scans. While other MRI researchers have designed specific scanning sequences to look at teeth in high detail, such scans are not readily available or used by most MRI studies. T1‐weighted scans, like those used in this study, are acquired as part of the majority of MRI research studies. Second, this study demonstrates that this information, once extracted, is actually useful in predicting behavior, in this case MoCA scores. Overall, this study suggests a possible pathway to researchers with MRI data that wish to explore the relationship between tooth count and various other measures of health or behavior.

### 4.1 Total tooth count and MoCA scores

Prior research indicates that oral/periodontal health can be used to predict cognitive status, including presence of early MCI, with converging evidence suggesting that tooth count is a particularly powerful predictor of cognitive decline. For example, one recent study reported that tooth count was associated with lower baseline cognition, 11‐year cognitive decline, and 15‐year dementia risk.^38^ And a recent meta‐analysis concluded that tooth count significantly impairs cognition and suggested that this may be a direct result of loss of mastication‐related sensory stimulation.^39^ Results from our study are consistent with prior findings in indicating that total tooth count and cognition are positively correlated. In the current study, this was true even after controlling for demographic differences, suggesting that total tooth count accounts for unique variance in MoCA scores. Interestingly, individual responses on oral/periodontal health questions did not contribute to predicting MoCA scores, suggesting that these questions may not be as useful as tooth count in relatively small samples. We would note that dental questionnaires may easily be modified to report “self‐assessed” tooth count; this may be a cost‐effective way to gather this meaningful data along traditional measures of oral/periodontal and periodontal health. We would also like to make the point that many large‐scale investigations of cognition did not initially acquire this data, and MR image inspection provides a powerful approach to retrospectively gather oral/periodontal health metrics. Finally, our results are important as they demonstrate the feasibility of deriving reliable measures of tooth count from research quality T1‐weighted MRI images, providing starting estimates for the percentage of participants for which valid measures of total teeth can be derived (i.e., rates of distortion in the ABC study's population were approximately 25% for the upper and lower jaw).

One interesting finding in the current study is that linear regression models with MoCA as the dependent variable could be significantly improved, above and beyond those based only on demographic and self‐reported oral/periodontal health metrics, when they included the MRI‐based measure of total tooth count. This claim should be interpreted in light of our findings when we split these data into healthy and at‐risk groups. We observed that, when considering only adults in the normal range for cognition (MoCA > 25, *N* = 160), total tooth count provided no additional predictive power. However, when considering adults with MoCA scores considered to be in the MCI range (MoCA <= 25, *N* = 36), we found that models including total tooth count were significantly better than those that did not include it.

While our data are consistent with broader findings relating tooth count and function to cognition,^38^, ^39^, ^40^ for example, a seminal study on this topic by Stein and colleagues found an association between low tooth count and incidence of dementia in 75–89‐year‐olds,^41^ it should be noted that our data do not offer a causal explanation for the observed association between tooth loss and MCI. Indeed, there is significant experimental evidence supporting the idea that the relationship between cognition and tooth loss is likely bidirectional; having impaired cognition leads to worse oral/periodontal care just as poor could lead to impairments in cognition.^42^ For example, loss of teeth can lead to impairments in mastication that impact the ability to eat healthy foods, such as fruits and vegetables, and this may lead to declines in health and cognition (Da^43^, ^44^). It is also clear that cognitive decline, especially when it involves memory loss, may impair oral hygiene and health. For example, failure to remember whether or not you have performed oral health‐related activities of daily living (ADLs) could result in decreased frequency of brushing and flossing. These examples only represent a fraction of the complex relationship between indirect measures of oral/periodontal health, such as total teeth and cognition. What does seem clear is that these two variables could form a vicious cycle that reduces the quality of life associated with aging, but with this thought, comes the counter idea that programs directed at minimizing loss of cognition and maximizing oral/periodontal health could be part of a virtuous solution.

### 4.2 Brain age gap (BAG) and MoCA Scores

Substantial evidence suggests that the difference between chronological age and BAG estimates generated by the brainageR algorithm (i.e., BAG) is associated with cognition in both healthy and clinical populations.^24^ Based on these reports, we expected BAG would be correlated with MoCA scores and that inclusion of BAG in linear regression models with MoCA as the dependent variable would significantly improve model fit. As expected, we report a strong relationship between BAG and MoCA scores in the expected direction. Individuals with older looking brains scored lower on the MoCA. Unexpectedly, inclusion of BAG in linear regressions predicting MoCA failed to significantly improve model fit after accounting for demographic variability. This suggests that much of the variability in brain age gap in the current study was already accounted for by variance in other demographic measures. In particular, we observed robust correlations between BAG and both race and age. This makes sense in light of prior literature showing a relationship between demographic variables and BAG.^45^, ^46^ Of course, there are a number of potential reasons why BAG might have failed to improve predictive models of MoCA scores. For one, our sample was relatively small compared to some larger‐scale studies that have been conducted to validate the utility of BAG in predicting cognition and health status. Larger studies like the one conducted here might better examine the differential utility of MR‐based dental and brain health information in predicting MoCA scores.

### 4.3 Automated methods of tooth enumeration for MRI

In the current study, tooth count was manually derived by visual inspection of research‐quality MRI data acquired as part of a larger investigation of aging. Manual enumeration of teeth, while not extremely time‐consuming, did require approximately 5 min per participant in the current study (x2 independent raters). This time includes opening the image and scrolling through a sufficient number of axial slices to confirm an accurate count of teeth in each quadrant as well as identify and log any MR distortion. In many cases there was no ideal axial slice that clearly showed all of the teeth in the upper or lower jaw. Accurate counting required raters to slowly scroll back and forth through axial slices using MRIcroGL12 (https://github.com/rordenlab/MRIcroGL) and “following the contours” of suspected teeth before confirming their presence. However, this manual approach may not be ideal for large‐scale databases which can contain thousands of MR images. One potentially promising avenue of future development would be to develop automated methods of extracting tooth count from standard research, or even clinical quality MR data. We suggest that researchers would benefit from the development of a valid and reliable pipeline for automating tooth count. Based on the current manuscript, models may need to be based on greater than one axial slice for both the upper and lower teeth. Possibly, models that extract 3D teeth from 3D NIFTI volumes would be even more accurate. At any rate, manual counts from trained raters will be needed in order to train AI or deep learning models to accomplish this task.

### 4.4 Implications for currently available open‐source brain imaging databases

**FIGURE 2:**
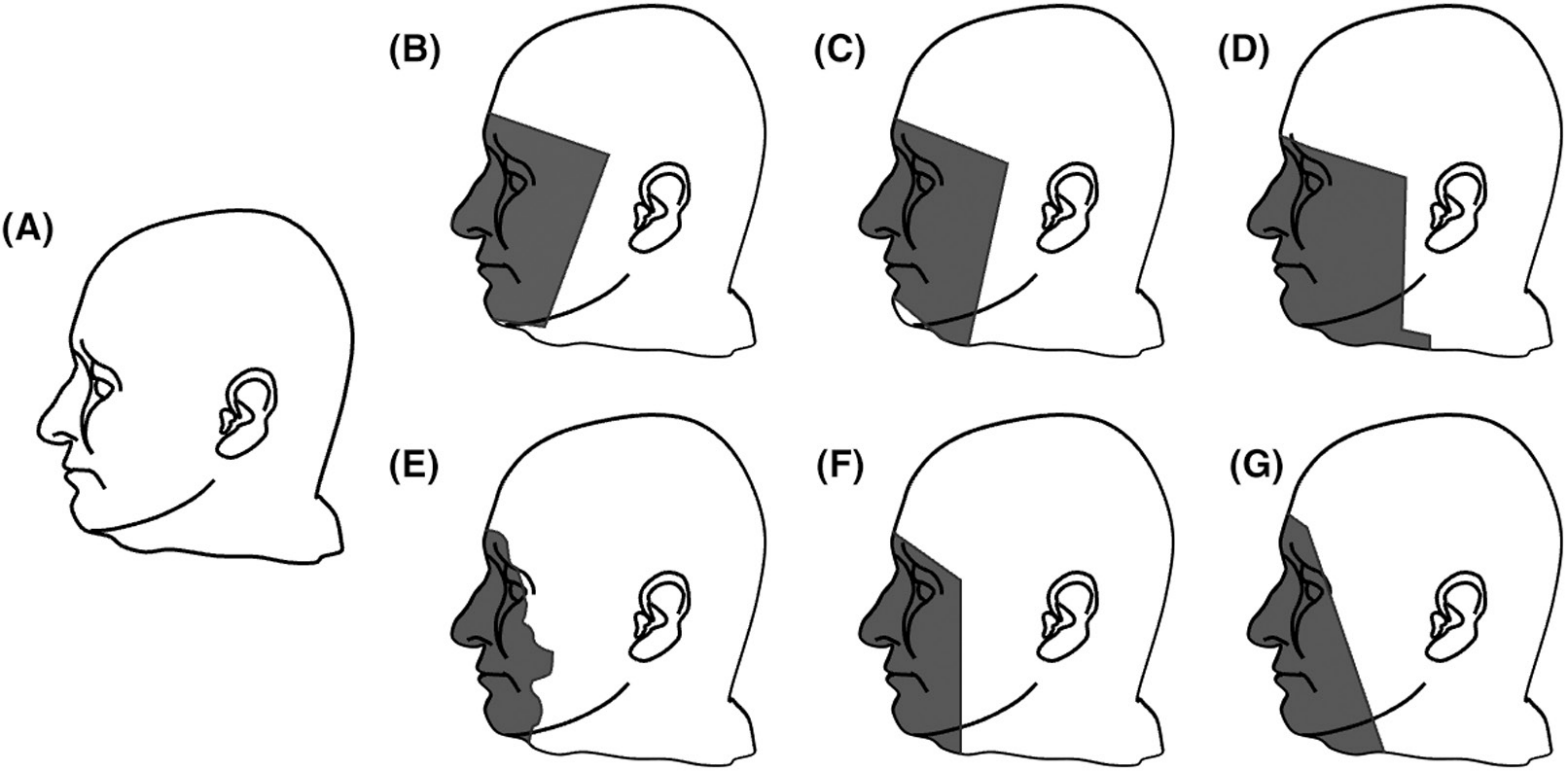
Public sharing of brain imaging data commonly requires that images be deidentified prior to sharing. The deidentification process involves removal of anterior identifying information including eyes, ears, nose, and jaw, and in some cases the ears. (A) Original image. Estimated cropping of images defaced using different currently available software are also shown for *afnirefacer* (B), *deepdefacer* (C), *mrideface* (D), *mridefacer* (E), *pydeface* (F), and *quickshear* (G).

Our findings have implications for the way in which brain imaging data is organized and shared. Large repositories housing brain imaging data typically constrain their organization according to the Brain Imaging Database Structure (BIDS) standard.^47^ This standard defines the way in which medical images should be organized. Major online imaging repositories additionally require that researchers thoroughly deidentify their medical images before uploading them. Specifically, PHI must be removed from image headers and brain images must be defaced, a process whereby identifying characteristics of the head (ears, eyes, nose, jaw) are removed/cropped. All currently available defacing algorithms remove the jaw and teeth (Figure 2), thus precluding counting of teeth as well as evaluation of other potentially meaningful dental metrics. The current findings make a strong argument that image locations containing potential oral/periodontal health information should not be stripped out of brain imaging data as it can provide potential value to predictive models of cognitive impairment. While teeth could be used to identify participants/patients, researchers must weigh the relative risks of defacing against the potential benefits gain of including relevant metrics that can be derived from them.

### 4.5 Limitations and future directions

This study has a number of limitations and caveats to interpretation. First, our usable tooth count data were reduced due to distortion present in MRI images. This is a major limitation as it limited the data we could use, and may have biased the results due to exclusion of these participants. Studies looking to adopt a similar approach should consider the fact that a certain percentage of MRI images will be usable for tooth count. A major weakness of this study is that oral/periodontal health questionnaire data was not collected from all participants due to the late introduction of this questionnaire into the ABC study protocol, thereby limiting the sample size for this analysis, and potentially biasing results. This likely limited our power to identify significant relationships between individual items on the oral/periodontal questionnaire and MoCA scores. A clear avenue for future research is to confirm the present findings in a larger clinical sample, such as the ADNI Alzheimer's Disease database (adni.loni.usc.edu). Additionally, our weak findings regarding the relationship between individual oral/periodontal health questionnaire responses and MoCA scores should not be interpreted as meaning that these two variables are unrelated. The sample size in this study was not comparable to the sample size of prior studies (*N* in the thousands) that report significant relationships between oral/periodontal health questionnaire responses and cognition.^48^, ^49^, ^50^ While we did find significant correlations between MoCA scores and tooth count, these correlations could have been different if the sample size had been larger.

Another limitation is that we relied on prior research on the relationship between MCI and MoCA scores to classify our MCI and nonMCI groups. It would be preferable to have a thorough assessment and diagnosis by a medical doctor. Furthermore, we only examined the utility of a single proxy of oral/periodontal health (tooth count) derived from structural MRI scans, and a single, widely used proxy of brain health (BAG) derived from the same scans. Other potentially informative biomarkers could be derived from structural or other imaging modalities. Regarding oral/periodontal health, while we focus on tooth count in the current study, it is important to consider that we do not know what the cause of the tooth loss was. There is ample evidence that tooth loss reflects overall oral/periodontal health.^51^, ^52^ For example, tooth loss could be caused by poor diet, poor dental care, lack of timely dental visits, and/or injury. Knowing the cause of tooth loss may provide additional information to researchers. While we controlled for basic sociodemographic variables in this study (age, sex, race, and SES), it should be noted that we did not control for multiple potential alternative confounders that could potentially explain variability in our data. Larger studies, with the explicit goal of creating comprehensive models of the relationship between oral/periodontal health and cognition, would be better able to control for larger numbers of potential confounding variables. A final limitation of this study is the high amount of data that could not be included for analysis due to distortion induced by metal. In cases where the MRI distortion in the mouth occluded other teeth, it was impossible to make a truly accurate tooth count. It is true that single implants cause different patterns of spatial distortion than, say dental bridges. Identification and classification of different types of distortions could provide richer data regarding oral/periodontal health. Exploring the relationship between specific types of distortion observed in MRI scans and actual dental x‐rays could be one way to explore this issue; such investigation could also benefit the development of automated tooth counting algorithms.

We would like to point out that a potentially promising future extension of our general approach would be to use existing medical images of the head to extract other metrics of dental health that could also potentially predict cognitive status. For example, it may be possible to quantify overjet or metrics related to tooth alignment (how twisted teeth are). More fine‐grained measures, like dental caries and amount of decay currently rely on high‐resolution (sub‐millimeter) medical images of the jaw,^53^, ^54^ but rapid advances in machine learning algorithms, may provide a way to extract these data from lower resolution data in the future. It is possible that these additional oral and/or periodontal metrics could provide even more predictive power to studies aimed at identifying MCI in older populations, particularly those at risk of converting to AD or dementia. The promise of automated algorithms for identifying such measures could yield other potentially useful markers for predictive models of MCI.

## 5 CONCLUSIONS

Overall, our data provide novel evidence regarding the relative utility of a relatively simple MRI‐derived metric of total tooth count, a proxy for oral/periodontal health in assessing cognitive function. Our simple demonstration that inclusion of tooth count data, but not BAG, in predictive models improves their accuracy provides a compelling argument for researchers interested in MCI and dementia to consider including total tooth count data in their models. These results highlight the value of considering oral/periodontal health, even when it can only be assessed indirectly, when modeling MCI, and have implications for the way medical images typically acquired as part of brain imaging experiments are distributed. It is suggested that future studies examine the relative value of MRI‐derived this and other oral/periodontal‐health metrics in both identifying individuals with MCI as well as predicting conversion to AD and dementia in at‐risk populations, and develop automated methods for calculating metrics like tooth count using large, open‐source medical image databases that traditionally focus on brain health.

## FUNDING INFORMATION

University of South Carolina (UofSC) Excellence Initiative, Aging Brain Cohort (ABC) Project.

## CONFLICT OF INTEREST STATEMENT

Roger Newman‐Norlund, Santosh Kudaravalli, Anwar Merchant, and Chris Rorden do not report any conflicts of Interest.

## Supporting information


Tables S1–S5

## Data Availability

The datasets generated and/or analyzed during the current study are available from the corresponding author upon reasonable request. Interested researchers can contact the corresponding author, Roger D. Newman‐Norlund, at rnorlund@mailbox.sc.edu to request the data.
