# Supplementary material for: Exploring the link between tooth loss, cognitive function, and brain wellness in the context of healthy aging
Source: J Periodontal Res. 2024 May 6;59(6):1184–94. doi: 10.1111/jre.13280 (PMC11626696; doi:10.1111/jre.13280)
Supplement: Supplementary file 1 — Tables S1–S5 [file JRE-59-1184-s001.docx]

**Supplementary Table 1.**

**Oral/Periodontal health questionnaire used in the current study (N = 197)**

| Q1 | Do you think you have gum disease? |
| --- | --- |
| Q2 | Has a dental professional ever told you that you have lost bone around your teeth |
| Q3 | Have you ever had scaling, root planing, surgery, or other treatment for gum disease? |
| Q4 | Have you ever had any teeth that have biome loose by themselves without some surgery (not baby teeth)? |
| Q5 | How often during the last week did you use mouthwash or any dental rinse product? |
| Q6 | How often, during the last 7 days, did you use dental floss, tape or an interdental brush to clean between your teeth, other than just to remove particles between your teeth? |
| Q7 | How do you rate the health of your gums? |
| Q8 | During the past 3 months, have you noticed a tooth that does not look right? |

Supplementary Table 1. In the current study, periodontal health was assessed via a set of standard questions related to oral/periodontal health. Subsequent statistical tests considered responses to individual items (Q1-Q8) as described in the **Methods** section.

**Supplementary Table 2.**

**Comparison of Linear Regression Models Containing Demographic Data versus Demographic Data plus Total Teeth (N = 197)**

**
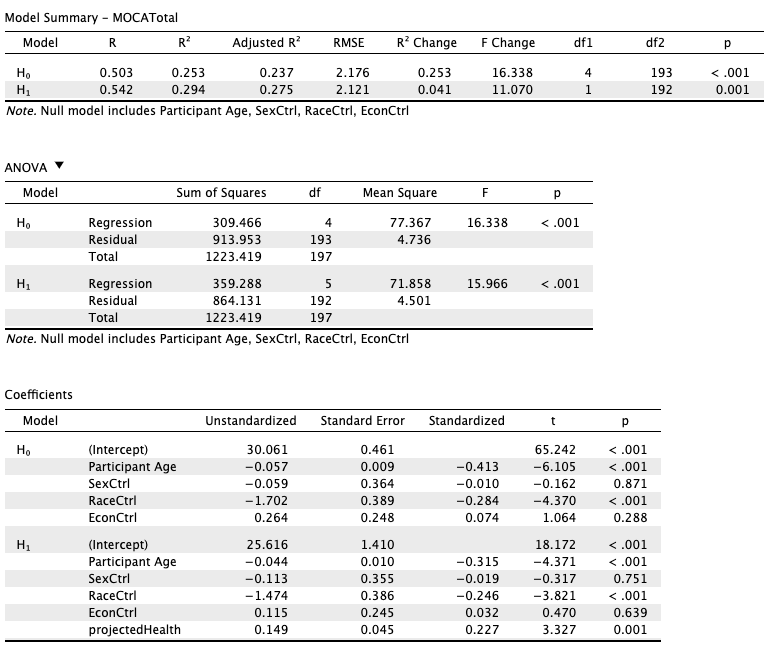
**

Supplementary Table 2. An ANOVA comparing linear regression models of MoCA total score were compared in all participants. The H_DEM_ model contained demographic information (age, sex, race, SES) while the H_DEM+TEETH_ model additionally contained total teeth. Inclusion of total teeth significantly improved model fit.

**Supplementary Table 3.**

**Comparison of Linear Regression Models Containing Demographic Data versus Demographic Data plus Brain Age Gap (BAG) (N = 282)**

**
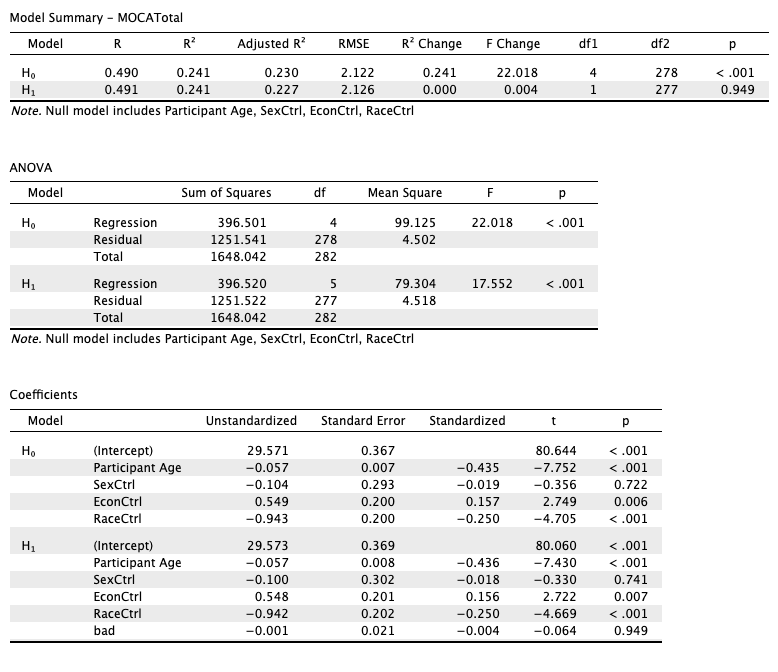
**

Supplementary Table 3. An ANOVA comparing linear regression models of MoCA total score were compared in all participants. The H_DEM_ model contained demographic information (age, sex, race, SES) while the H_DEM+BAG_ model additionally contained brainage gap (BAG). Inclusion of BAG did not significantly improve model fit.

**Supplementary Table 4.**

**Comparison of Linear Regression Models Containing Demographic Data versus Demographic Data plus Total Teeth in MCI group (N = 36)**

**
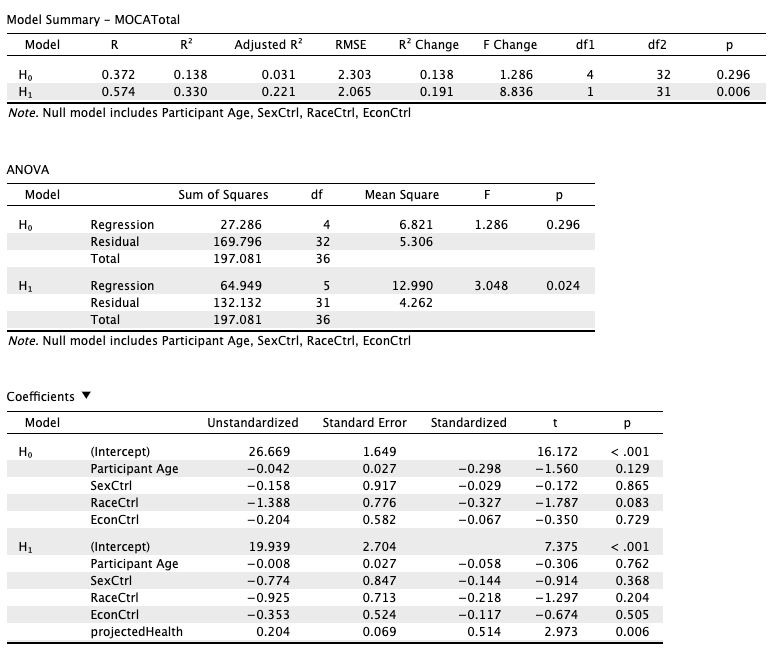
**

Supplementary Table 4. An ANOVA comparing linear regression models of MoCA total score were compared in a subset of participants with MoCA scores <=25 (MCI group, N = 36). The H_DEM_ model contained demographic information (age, sex, race, SES) while the H_DEM+TEETH_ model additionally contained total teeth. Inclusion of total teeth significantly improved model fit.

**Supplementary Table 5**

**Comparison of Linear Regression Models Containing Demographic Data versus Demographic Data plus Total Teeth in non-MCI (N = 160) group**

**
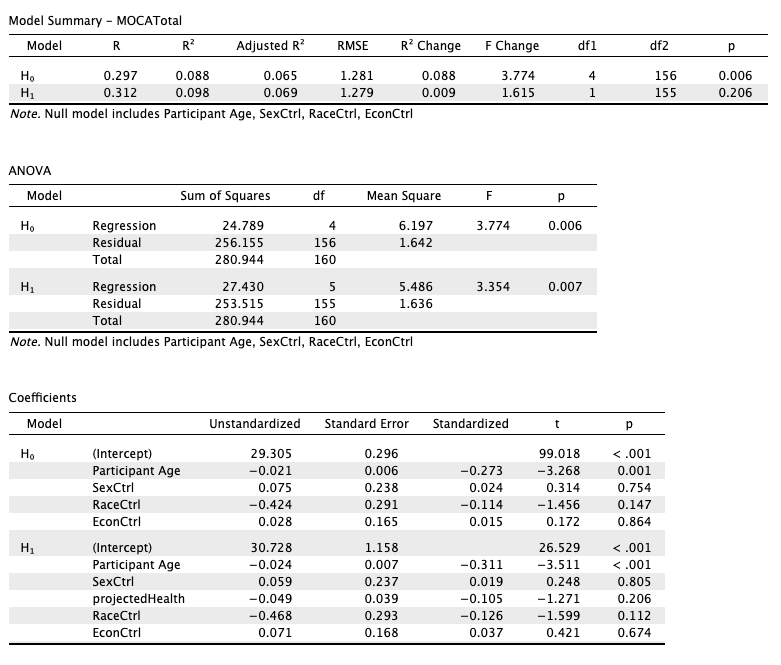
**

Supplementary Table 5. An ANOVA comparing linear regression models of MoCA total score were compared in a subset of participants with MoCA scores >25 (healthy non-MCI group). The H_DEM_ model contained demographic information (age, sex, race, SES) while the H_DEM+TEETH_ model additionally contained total teeth. Inclusion of total teeth did not significantly improve model fit.
